# A tribute to Paolo Pelosi

**DOI:** 10.1186/s13054-023-04521-7

**Published:** 2023-06-27

**Authors:** Chiara Robba, Denise Battaglini, Lorenzo Ball

**Affiliations:** 1 Department of Surgical Sciences and Integrate Diagnostics, University of Genoa, Viale Benedetto XV 16, Genoa, Italy; 2 Anesthesia and Critical Care, IRCCS San Martino Policlinico Hospital, Genoa, Italy


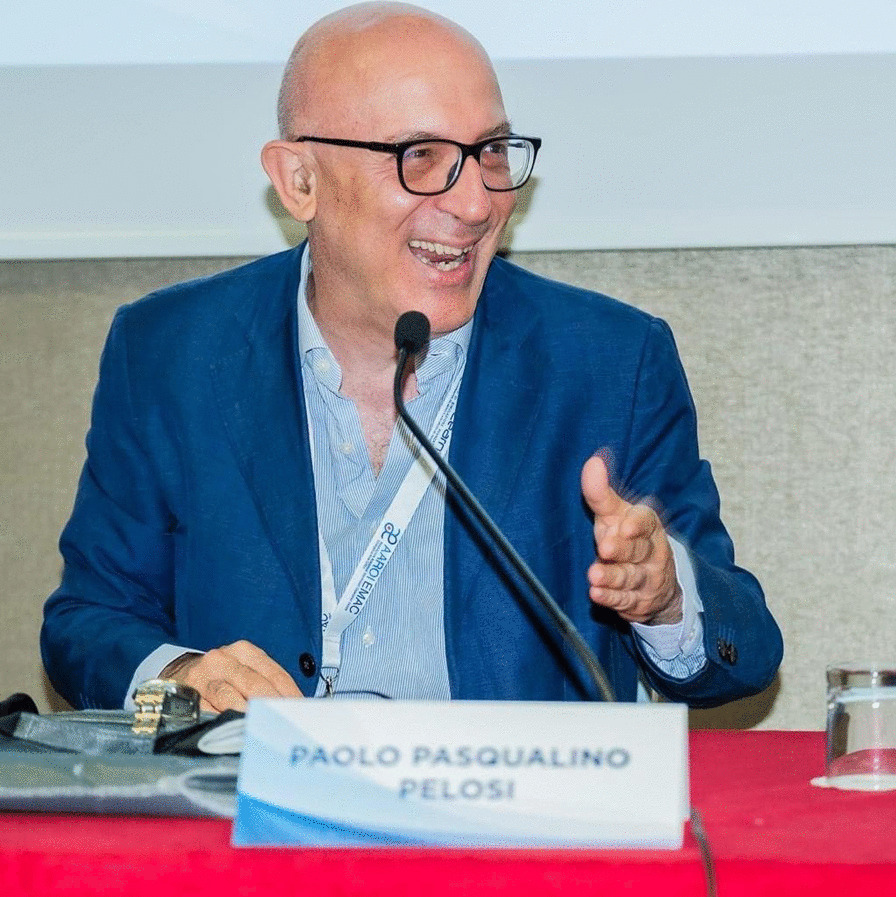


On the 30th of May, Professor Paolo Pelosi passed away. The critical care community from all over the world is mourning his death. Professor Pelosi was a leader in the field of anaesthesiology and intensive care medicine, with his continuous contributions to education, research, and clinical practice. He was Chief Professor in Anaesthesia and Intensive Care at San Martino Policlinico Hospital in Genoa, Italy, and Director of the Speciality School in Anaesthesia and Intensive Care at the University of Genoa.

He was extremely active and was an influential figure internationally. Prof Pelosi served as President of the European Society of Anaesthesiology (ESA) from 2010 to 2011, and had important roles in the World Federation of Societies of Intensive Care and Critical Care Medicine (WFSCCM) and European Respiratory Society (ERS). He was the vice President and future president (2025–2027) of the Italian Society of Anesthesia Analgesia and Intensive Care (SIAARTI). His contributions were further recognised through honorary memberships in several scientific societies of Anaesthesiology and Intensive Care worldwide.

His last achievement was the prestigious “International Career Award in Intensive Care Medicine from Chilean Society of Critical Care and Emergency Medicine-SIEMPAC”, just a few days before dying.

He has been speaker, and organizer of many scientific congresses. He served for many years as Associate Editor for Critical Care, and in the editorial board of several other journals. He had been a scientific advisor of the annual International Symposium of Intensive Care and Emergency Medicine (ISICEM).

In every role he covered in national and international organisations and societies related to Anaesthesiology and Critical Care, he has always been an influential figure providing enthusiasm and passion for the advancement and progress of the medical community. He has always tried to look beyond the limits and to push forward the current knowledge, creating strong groups and collaborations based of friendship and science.

His open-minded vision, his willingness of improving knowledge and of providing honest intellectual messages has profoundly changed the way of all physicians to treat patients through his numerous lectures, and scientific contributions. Professor Pelosi made the difference in the field of mechanical ventilation and acute respiratory distress syndrome (ARDS), in particular through the work of the PROVEnet group, which allowed to create large-scale observational studies and randomized clinical trials, providing high quality evidence which revolutionised the field of anaesthesiology and intensive care medicine. During the COVID-19 pandemic, his faithful commitment to the work has importantly helped the scientific community to face this difficult moment.

Professor Pelosi will be remembered for his intellectual contribution and leadership in the field. But we—as part of his research group—had the privilege to collaborate with him on a daily base over the last years in Genoa, and we have been lucky enough to appreciate the man behind his extraordinary curriculum. He built our team, day by day, pushing his collaborators to constant improvement, to achieve scientific independence and to never surrender. He gave us the possibility to grow up as scientists and persons. He taught us that hard work and passion are the only way to achieve results in our work, he helped us to believe in ourselves and to think big, with honesty and loyalty. He always advised us to take the road uphill to achieve success.

He was a noisy, funny, self-mocking mentor, colorful in every facet of his being. He was an extraordinary hard worker, and a brilliant mind. His profound religious faith has always accompanied him in his work and has given him strength in the last, very difficult months in which he had to face the most difficult challenge of all, the one against his disease. His personal beliefs were always put in a context of interreligious and intercultural openness and acceptance. Even in the last days before passing, he was serene, hopeful and supportive with all of us.

We all have lost a renowned scientist, a colleague, a loyal friend, a mentor.

Our thoughts are with his family and close friends, especially his son, his wife and his mother, but also to all the medical community worldwide who has been close to him.

His motto, every time we concluded a project, was *“To the next”,* which clearly describes his constant wiliness to move on and never stop. And this is what we are going to do, keeping his teaching in our hearts.

Arrivederci, Prof. You are missed and loved.

It was a privilege working with you: we will never forget you.

*“Let’s climb the mountain together”*, as always.

## Data Availability

Not applicable.

